# Selenium reduces oxaliplatin induced neuropathic pain: focus on TRPV1

**DOI:** 10.3389/fphar.2025.1549190

**Published:** 2025-03-07

**Authors:** Bilal Çiğ

**Affiliations:** Kirsehir Ahi Evran University Medicine Faculty Department of Physiology, Kirsehir, Türkiye

**Keywords:** selenium, TRPV1, oxaliplatin, OX-IN, oxidative stress, ROS

## Abstract

Many drugs preferred for pain relief are insufficient against oxaliplatin (OX) induced neuropathic pain (OX-IN). Studies have shown that such pain mediators as the TRPV1 channel play a critical role in triggering high-sensitivity pain response in the dorsal root ganglia (DRG). TRPV1 activated by oxidative stress increases cytosolic free Ca^2+^ levels and leads to apoptotic cell damage. The key factors involved in the pathophysiology of OX-IN, which involves many components, are mitochondrial dysfunction and oxidative stress, both triggered by excessive Ca^2+^ influx across the neuronal membrane. Selenium (Se), an essential trace element, prevents the harmful effects of this oxidative stress through glutathione peroxidase. This study is based on understanding the neuroprotective role of Se, a cofactor for glutathione peroxidase, against TRPV1-mediated oxidative damage, mitochondrial dysfunction and apoptosis in OX-IN using molecular techniques such as patch clamp. The primary target in this study was DRGs as the initial station of OX-induced peripheral pain isolated in adult rats. In addition to the SN (sciatic) neurons isolated from the same animals, *in vitro* breast cancer cell (MCF-7) was also used to confirm the results. The study was conducted with four groups: control (5% dextrose), OX (4 mg/kg OX twice a week), Se (1.5 mg/kg Se every other day) and finally OX + Se, all of which were administered to the animals intraperitoneally for 4 weeks. The OX (50 μM for 24 h) and Se (200 nM for 2 h) were applied to MCF-7 cells *in vitro*. Although an excessive increase was observed in Tumour necrosis factor α (TNF-α), interleukin-1β (IL-1β), and interleukin-6 (IL-6), as well as mitochondrial oxidative stress, apoptosis and TRPV1 channel overactivations in DRG and SN neurons under OX treatment, Se suppressed these negative effects. While OX reduced glutathione peroxidase and significantly increased malondialdehyde level (LP) in DRG neurons, Se reversed this situation. In conclusion, the TRPV1-mediated efficacy of Se in suppressing OX-induced pain symptoms was demonstrated and we concluded that Se should be considered in future therapeutic approaches in the treatment of OX-IN.

## Introduction

Oxaliplatin is a widely used first-line chemotherapy agent for many types of tumors, including colorectal carcinomas (Raymond et al., 1998), although neuropathic pain is a common side effect of such chemotherapeutics (Wolf et al., 2008). OX-IN often causes paresthesia in the extremities (distal), in addition causes loss of coordination and balance due to peripheral sensory damage (Price et al., 2008). Such pain limits the use of treatments involving potentially effective anti-cancer drugs (Connelly et al., 1996), and although analgesics such as gabapentin, which are widely used for the treatment of neuropathic pain, have been shown to be inadequate for OX-IN (Sisignano et al., 2014), these ineffective therapies highlight the need for alternative treatment options based on molecular mechanisms rather than symptomatic therapies. OX-IN causes a serious increase in calcium (Ca^2+^) ion levels. Ca^2+^ plays a vital role as a secondary messenger in the modulation of many cellular functions. While this important tasks, excessive intracellular calcium [Ca^2+^]_i_ accumulation can lead to serious problems in OX-IN pain. Excessive abundance of [Ca^2+^]_i_ over time can damage cellular physiological functions, increase the production of reactive oxygen species (ROS), and lead to mitochondrial dysfunction (Nazıroglu, 2009). In addition, a study reported that Ca^2+^ ions mediated cell damage in a DRG culture (Sun and Windebank, 1996). Moreover, chemotherapeutic agents trigger neuropathic pain in DRGs by altering the expression levels of some ion channels localized in the plasma membrane, causing excessive changes in the cytosolic ionic environment and [Ca^2+^]_i_ (Jaggi and Singh, 2011). TRP superfamily, one of these ion channels, play an extremely important role in the [Ca^2+^]_i_ change in OX-induced OX-IN. OX contributes to the reduction of activation threshold in DRG neurons, particularly due to the sensitivity of TRPV1’s from the vanilloid subfamily (Anand et al., 2010). TRPV1, the main focus of our study, has made significant contributions to neuropathic pain research to date (Materazzi et al., 2012), is a tetrameric capsaicin (CAP) and proton-sensitive cationic receptor that promotes peripheral noxious thermal stimuli and is activated by painful chemical stimuli and inflammation (Caterina et al., 1997; Xiang et al., 2017). It is well expressed in peripheral neurons, including DRGs, and contributes significantly to pain and inflammation caused by various compounds, including irritating chemicals and reactive oxygen species (Hu et al., 2019).

It is well known that the numerous TRP channels processed in several cell types use cysteine residues to detect changes in redox states. TRPV1 has been identified as the primary mechanism behind cysteine oxidation in response to redox changes, but this has yet to be fully elucidated biochemically (Ogawa et al., 2016). Se, a trace element with important biological roles, plays an important role in proteins through its selenomethionine and selenocysteine structures (Balaban et al., 2017). Although it is well known that Se provides neuroprotective effects against oxidative stress in mammals (Rayman, 2000), its roles mediated by TRPV1 channels in OX-IN have not yet been revealed. Similarly, its role in molecular processes such as ion channel dysregulation, mitochondrial dysfunction, oxidative stress and apoptosis in OX-IN is not yet understood sufficiently This study makes a significant contribution to the understanding of the TRPV1-mediated protective role of Se against OX-IN. Here we induced OX-IN pain conditions and then investigated the TRPV1-mediated neuroprotective effects of Se on cytosolic Ca^2+^ levels, apoptosis, oxidative stress, etc. in rat DRG and sciatic neurons as well as *in-vitro* MCF-7 cells.

## Materials and methods

### Chemicals

OX, Celdach (100 mg) was sourced from India (Flat No. 203, Moksha Castle, Kalyan Nagar, Venture No. 3, Hyderabad, Telangana, 500018, India); sodium selenite 99% was sourced from sigma Aldrich; and Fura-2 AM was obtained from Thermo Fisher Scientific in the United States (81 Wyman Street, Waltham, MA 02451).

### Animals

For the study, 40 female Wistar Albino rats (12 weeks old, 180–200 gr) were obtained from the Suleyman Demirel University DEHATAM Experimental Animals Unit. The rats were kept four or five rats to a cage, illuminated from morning until evening (08:00–20:00, 12-h light/dark cycle), in special rooms under room conditions (temperature 25°C, humidity 65%–70%). The animals were fed with specially formulated pellet diets containing essential nutrients for their growth and health. The feeds produced for rats are cylindrical brown dry pellets. Their content consists of corn, soy, wheat and various vitamin-mineral mixtures. These feeds are generally prepared to meet all the nutritional elements required for the healthy growth and biological functions of rats in their cages in the experimental animal unit.

### *In vivo* study design

**FIGURE 1:**
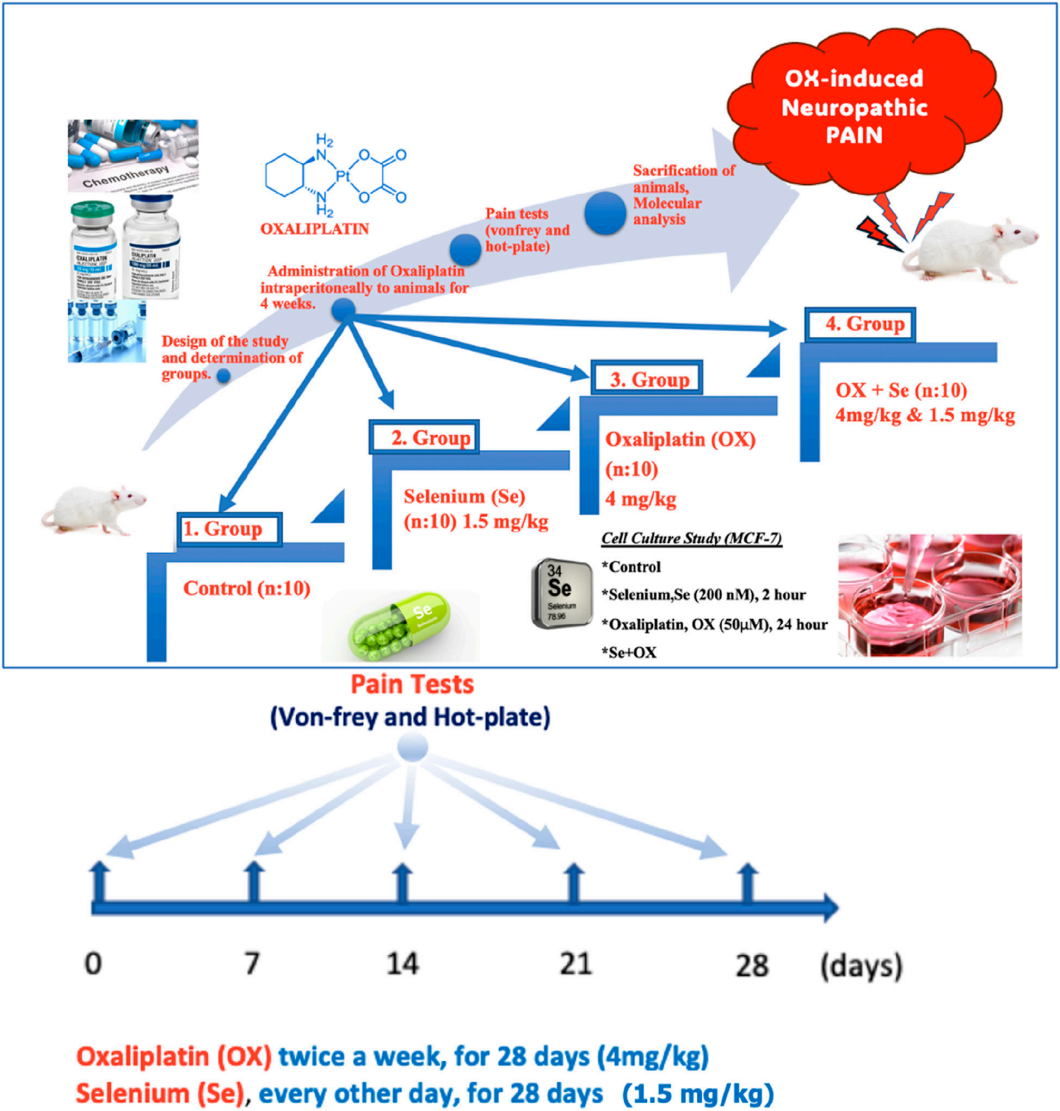
Schematic representation of the study. OX-exposed animals were treated with Se every other day and their effects on OX-IN were investigated by molecular level analysis. These groups were also formed *in vitro* in the MCF-7.

The rats were divided into four groups for the *in vivo* stage of the study:

Control (n = 10): The rats underwent no drug treatment, being administered 1 mL of 5% dextrose intraperitoneally once a day as a placebo for 28 days (Figure 1).
Se (n = 10): The animals in this group were administered Se intraperitoneally (on alternate days for 28 days, 1.5 mg/kg) (Kahya et al., 2017) (Figure 1).
OX (n = 10): The animals in this group were administered OX intraperitoneally (4 mg/kg twice a week for 28 days) (Fujita et al., 2015) (Figure 1).
OX + Se (n = 10): The animals in this group were administered OX (twice a week, 4 mg/kg) and Se (1.5 mg/kg, on alternate days) intraperitoneally for 28 days (Figure 1).

The OX and Se were dissolved in 5% dextrose for administration to the animals (i.p.) (Fujita et al., 2015). These animals in all groups except the control were given 5% dextrose on the days they did not receive the drug (Se and/or OX).

### Pain threshold tests

The OX-IN was measured using different thickness Von-Frey filaments (Aesthesio, Japan). The responses to needles of different thicknesses designed, specifically for the pain test, were determined from the animal’s retraction of the hind paw. A temperature-controlled plate (Variomag, Germany) was used for the hot-plate test, evaluating the paw withdrawal threshold time for thermal nociceptive stimuli. The rats were placed on the hot plate device, the surface temperature of which was 55°C, and the reaction time recorded (Kahya et al., 2017).

## Preparation of DRG and SN neurons

Following anaesthesia (ketamine and xylazine, 100 mg and 12.5 mg/kg, respectively), the animals were decapitated (for DRG isolation), and the vertebral column was dissected immediately. The vertebralis column was separated from the muscle tissue with the help of a scalpel and microsurgical scissors, and divided symmetrically into two equal parts, from the cervical to caudal levels. The spinal cord was held with forceps and removed. All these operations were carried out on ice. Dorsal root ganglia (from Thoracal13 to Lumbar5) nodules located in the intervertebral regions were collected one by one under fluorescent microscope and transferred into a DMEM medium. The ganglion nodules were subjected to enzymatic and mechanical dissociation processes to break down the rigid connective tissues and to obtain DRG cells (Kahya et al., 2017). For the SN isolation, briefly, after the animals were anesthetized as above, the SN was removed from the hind limb through blunt dissection along the biceps femoris, and the removed nerve was placed in a DMEM medium. Mechanical digestion was achieved with the help of microsurgical scissors, while enzymatic digestion was achieved using a trypsin enzyme.

### *In vitro* MCF-7 cell culture study (planned in four groups)

MCF-7 (ATCC, HTB-22) breast cancer cells from cell lines in which TRPV1 channels were expressed were used for the cell culture analyses. The groups were as follows:

Control group (n = 6): The cells were taken into the cell culture medium for 24 h without being treated with any medication and then incubated for 2 h with saline (Figure 1).
Se group (n = 6): The cells were treated with 200 nM Se for 2 h after being kept in the cell culture for 24 h (Figure 1) (Uğuz and Nazıroğlu, 2012).
OX group (n = 6): This group was treated with OX (50 µM) for 24 h and then incubated with saline for another 2 h (Figure 1) (Ghazizadeh and Nazıroğlu, 2014).
OX + Se group (n = 6): This group was treated with OX (50 µM) for 24 h and then incubated with 200 nM Se for an additional 2 h (Figure 1).

### Measurement of intracellular calcium Ca^2+^ signal analysis

The intracellular free calcium concentration level [Ca^2+^]_i_ was analysed with a Fura-2 a.m. calcium indicator (Kahya et al., 2017), and the contribution of the TRPV1 cation channels to changes in the intracellular calcium ion levels in OX-induced peripheral neuropathic pain (Cary Eclipsys, Varian Inc., Sydney, Australia) was evaluated. Changes [Ca^2+^]_i_ were recorded on a computer screen for approximately 450–500 s for excitation, emission wavelengths, 340/380 and 505 nm respectively (Uğuz and Nazıroğlu, 2012). The obtained values were replaced by the formula developed by Grynkiewicz (Grynkiewicz et al., 1985) and the intracellular calcium levels of the groups were evaluated.

### Electrophysiology (patch-clamp)

Electrophysiological current records were obtained from DRG neurons using a patch-clamp technique (EPC 10, HEKA, Germany). The current records obtained from the whole cell configuration were the records of the TRPV1 channel. We used specific TRPV1 agonists and antagonists to investigate the roles of these channels in OX-induced peripheral pain. The analysis and interpretation of the obtained data were carried out using the patch-master program on a personal computer. Borosilicate micropipettes (Novato, CA, United States), which we prepared with a puller device with a tip thickness of 1–2 μm, were placed in the pipette holder to contact the cells. By giving appropriate commands in x, y, and z coordinates, it was ensured that the appropriate cell was observed under the patch microscope, that the cell was contacted with the micropipette to provide suction, and that consequently, recordings were taken of the whole cell configuration. The cell resistances of whole cell recording electrodes were 4–7 MΩ. The holding potential was set at −60 mV, and current-voltage (I-V) relationships were obtained from voltage ramps of −90 mV to +60 mV, applied over 400 ms. The patch-clamp technique used here involves measuring changes in current by fixing the voltage and touching a single cell with the help of a micropipette. Detailed information on patch-clamp applications has been reported in the article by Kahya et al. (2017). CAP was applied (10 μM) in the bath chamber, and TRPV1 was then specifically inhibited through the application of 100 μM of capsazepine (Cpz) intracellularly (inside the pipette) and extracellularly (bath chamber). NMDG: N-methyl-D-glucamine. To ensure the viability of the recorded cell, an extracellular buffer solution with NMDG^+^ is sent to the chamber and the recording is continued. NMDG^+^ acts as sodium replacement. For analysis, the maximum current amplitude in a sample cell is found by proportioning to its capacitance value, which is directly proportional to the surface area of that cell. Experimental results are presented in the form of line graphs and current density (pA/pF) graphs of the original records.

### Determination of reactive oxygen species (ROS) analysis

ROS (Thermo Fisher Scientific, catalog number: EEA019) determination was measured using a multi-well reader (Infinite Pro200) (Excitation: 488 nm, emission: 543 nm) (Balaban et al., 2017). The data were evaluated and compared with the control group data.

### Mitochondrial membrane depolarization analysis

Samples were treated with mitochondrial membrane depolarization assay dye JC-1 (Thermo Fisher Scientific, catalog number: M34152) (1 μM, 37°C, 15 min) [17]. The fluorometric changes between groups were analysed using a multi-well reader (Infinite Pro200) (Excitation: 485 nm, emission: 535 nm). The data were evaluated and compared with the control group data (Yüksel et al., 2017).

### Apoptosis level analysis

The apoptosis test (APO Percentage apoptosis kit Biocolor) was applied as previously described (Akpınar et al., 2016). When the asymmetry of an apoptotic cell membrane disappears, the APO Percentage dye enters the cell, staining it red and allowing the determination of apoptosis in a spectrophotometric measurement. The data were expressed in fluorescence units/mg protein and compared with the control values.

### Cytokine level analysis

The TNF-α (cat. number: KRC3011), IL-1β (Cat. number: BMS630) and IL-6 (cat. number: ERA31RB) cytokine levels (Thermo Fisher Scientific) in DRG, SN and hippocampal neuron cells were determined using the Elisa kit method (Demirci et al., 2017). The cytokine quantities in the cells were analysed in multiple wells at 450 nm (Tecan Inc., Groedig, Austria). Cytokine levels were expressed in units of pg/mL for the different primary cell types.

### Analysis of lipid peroxidation (LP), glutathione (GSH) and glutathione peroxidase (GSH-Px) levels in DRG

The lipid peroxidation levels (LP) in DRG neurons were evaluated based on the reaction of thiobarbituric acid (TBA) (Sigma, MO, United States) for 20 min at 95°C. LP levels were determined by spectrophotometer (Shimadzu, Japan) at 532 nm. LP and GSH results were expressed as µmol/g protein, while GSH-Px was expressed as IU/g protein. The GSH determination was analysed at a 412 nm wavelength using an Ellman reagent (Nazıroglu et al., 2004). The GSH-Px was measured in accordance with the method described in a previous study. Using the Bradford method (Noble and Bailey, 2009), protein densities were measured at 595 nm with the aid of a Coomassie Plus reagent.

### Statistical analysis

An unpaired Mann-Whitney U-test and an analysis of variance (ANOVA) were performed (p was considered statistically significant for cases less than or equal to 0.05). A nonparametric test was used for simple observational pain tests (Von Frey and Hot plate). Statistically acceptable values were evaluated with a least significant difference test.

## Results

### Investigation of Se’s role in OX-IN through Von Frey and Hot-plate pain tests

**FIGURE 2:**
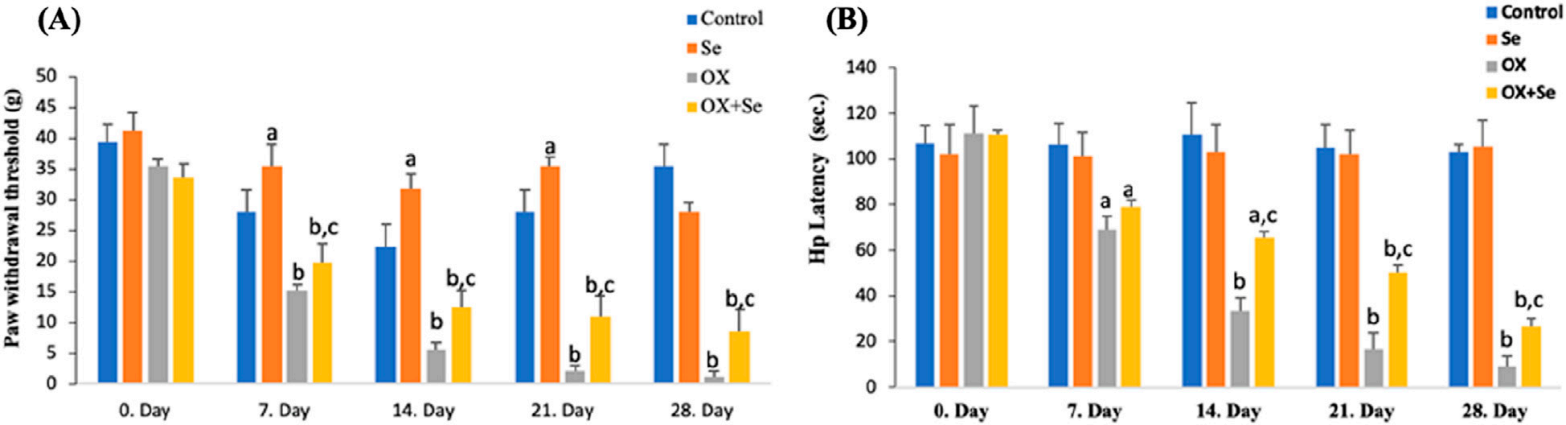
The effect of Se on 12-week Wistar-albino female rats treated with OX in terms of paw withdrawal threshold and latency tests (respectively Von frey, Hot plate). **(A)** This graph expresses the effect of Se-therapy on the withdrawal force in OX-treated rats. (^a^p ≤ 0.05, etc. than control; ^b^p ≤ 0.001, etc. than 0. Day and control groups; ^c^p ≤ 0.05, etc. than OX), (n = 10 and mean ± SD). **(B)** This graph shows the effect of Se on latency in rats exposed to OX (^a^p ≤ 0.05, ^b^p ≤ 0.001, etc. than 0. Day: ^c^p ≤ 0.05, etc. than OX), (n = 10 and mean ± SD).

We evaluated the effect of OX (4 mg/kg, for 28 days) on peripheral pain development in animals by examining paw withdrawal latency and paw withdrawal threshold values, based on Von Frey and hot-plate observational pain tests. Peripheral neuropathic pain was observed 1 week after the administration of OX to the animals and was evaluated through Von Frey and hot plate tests, as simple observational pain tests, during the twice weekly OX injections (28 days). Here, the aim was to reveal whether OX caused peripheral pain. The results were truly incredible, with significant reductions noted in paw withdrawal threshold and latency during the Von Frey (needle tips of different thickness) and hot plate (Figures 2A, B) pain tests of OX-injected rats (ip 4 mg/kg). After the seventh day, the decline continued gradually over the following weeks. An anti-hyperalgesic effect was observed when Se was administered (OX + Se) to the rats treated with OX, compared to the just OX groups (p ≤ 0.05; Figures 2A, B). In addition, Se was found to increase the sensitivity threshold (p ≤ 0.05; n = 10 and mean ± SD).

### Electrophysiology (patch-clamp) results in DRG neurons

**FIGURE 3:**
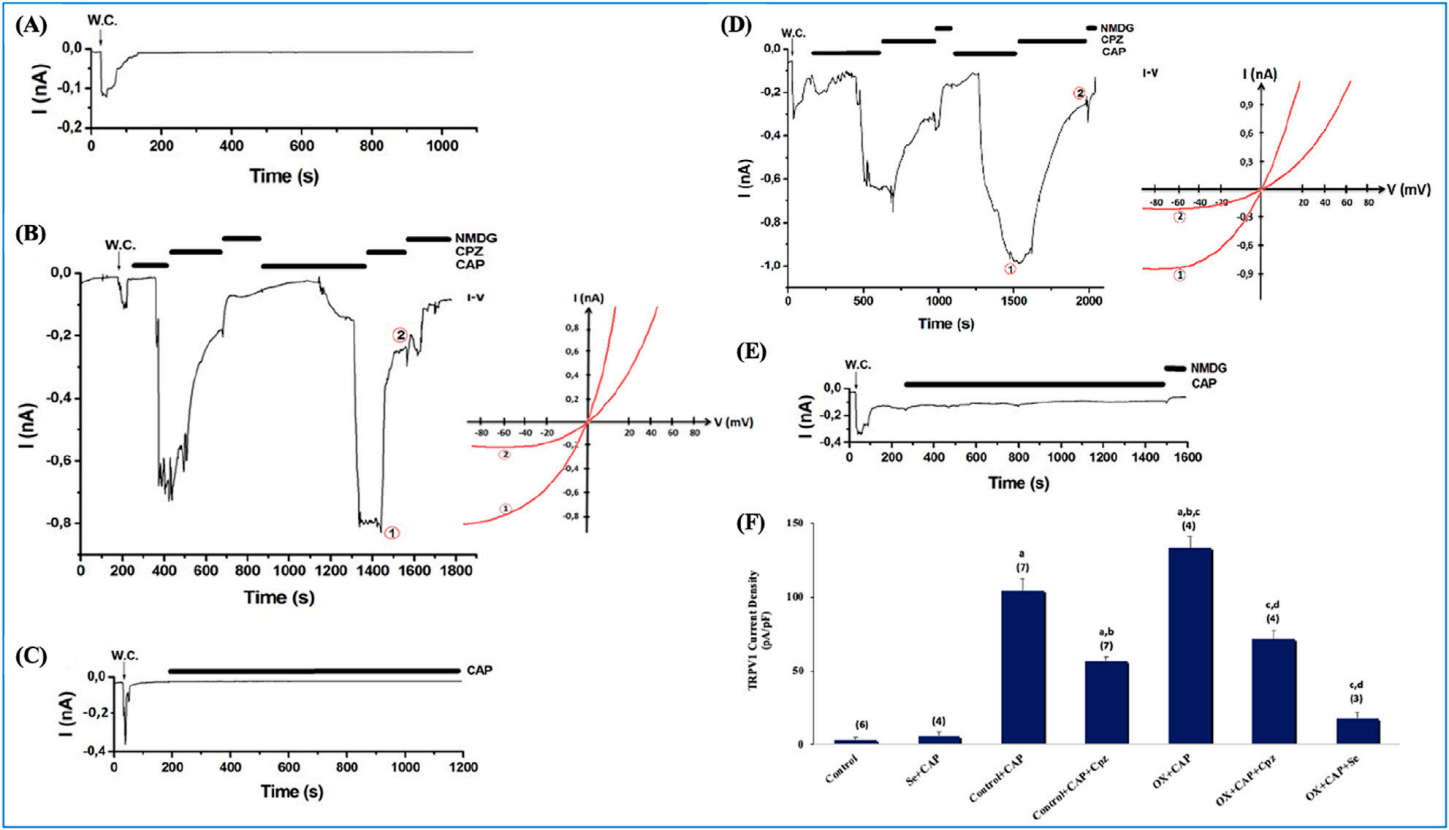
Current densities in DRGs isolated from OX and Se treated rats. The holding potential for these records was −60 mV. **(A)** Control (n = 6): These are patch-clamp records from rat DRGs that have not undergone any treatment. **(B)** Control-CAP group (n = 7): Unlike control, this group was stimulated with an agonist (TRPV1 specific agonist, CAP, 0.01 mM). It was also inhibited by Cpz (0.1 mM). **(C)** Se-CAP group (n = 4): DRGs of Se treated rats were stimulated by CAP. **(D)** OX-CAP group (n = 4): DRGs of OX treated rats were stimulated with CAP (TRPV1 agonist, 0.01 mM) using patch-clamp techniques. Inhibited by Cpz (0.1 mM). **(E)** OX-CAP-Se group (n = 3): TRPV1 currents were examined in the DRGs of OX and Se treated rats with using patch-clamp technique. CAP (0.01 mM) was used in stimulation and Cpz (0.1 mM) was used for inhibition. **(F)** In this graph, current densities in DRG cells were compared for all groups. n: this is the number of repetitions for each group (^a^p ≤ 0.00, 1 etc. than Control and Se; ^b^p ≤ 0.001, etc. than Control + CAP; ^c^p ≤ 0.001, etc. than CAP + Cpz; ^d^p ≤ 0.001, etc. than OX + CAP). I-V means current-voltage relationship. NMDG, N-methyl-D-glucamine.

The effects of OX on TRPV1 channels are presented in Figure 3. CAP (10 µM) induced a current in the DRG neurons. The curve graphs are the current records that develop after CAP is added to the patch chamber (Figure 3). All current records were taken in whole cell configuration. Figure 3A shows that no current was observed in the absence of CAP. Within this group, the cell was subjected to suction with a micropipette, and the recording configuration was switched from cell attached to whole cell. In Figure 3B, the recorded current value in the control + CAP group was approximately −800 pA (−60 mV holding potential). In Figure 3C, Se completely blocked the activation of the channel, and no current was observed, despite the administration of capsaicin. In Figure 3D, The OX + CAP group reached approximately −1,000 pA, which was the maximum value among all groups. As can be seen in Figure 3E, Se with OX was administered to the animals i. p. and no current was observed, despite the addition of CAP to the patch chamber. The current densities in DRG primary sensory neuron cells were significantly higher in the OX + CAP groups than in the control and control + CAP groups (p ≤ 0.001) (Figures 3A, B, D, F), and there was a significantly greater reduction in the Control + CAP + Cpz than in the Control + CAP group (p ≤ 0.001) (Figures 3B, F), and a significantly greater reduction in the OX + CAP + Cpz than in the OX + CAP group (p ≤ 0.001) (Figures 3D, F). In addition, Se suppressed channel activations significantly in the Se + CAP group when compared to Control + CAP (p ≤ 0.001) (Figures 3B, C, F); while in the OX + CAP + Se group, the reduction was significantly greater than in the OX + CAP group (p ≤ 0.001) (Figures 3D–F).

### Ca^2+^ signal in painful (OX-IN) Rat’s DRG and SN neurons

**FIGURE 4:**
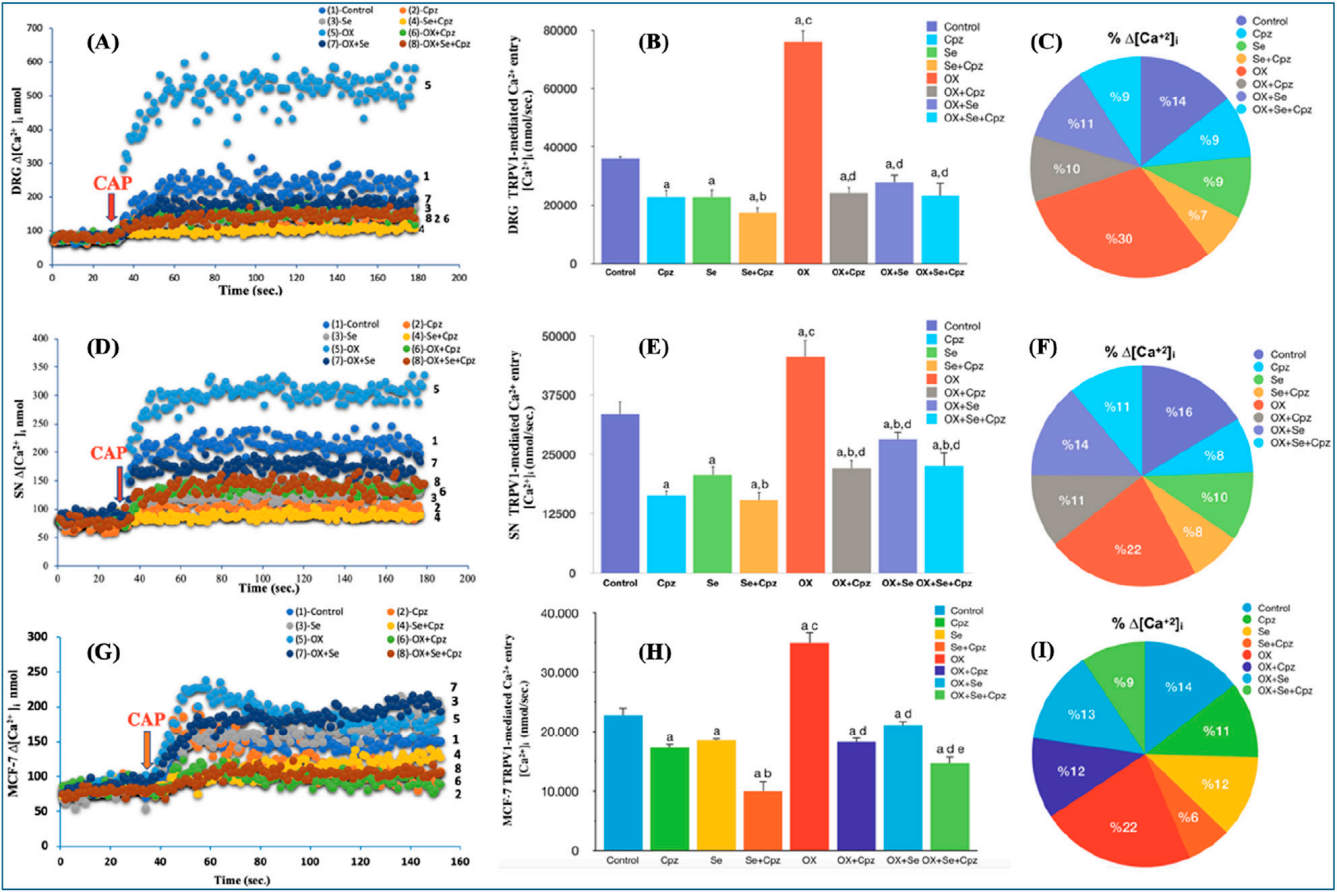
Se treatment mediated change in [Ca^2+^]_i_ through TRPV1 ion channels in OX exposure in DRG, SN neurons and MCF-7 breast cancer cells. Stimulation was performed with CAP (0.1 mM CAP). **(A)** The time-dependent change in DRG cells in CAP-stimulated [Ca^2+^]_i_ was expressed for all groups. **(B)** Effect of Se on TRPV1 mediated [Ca^2+^]_i_ in OX-exposed DRG cells (^a^p ≤ 0.001, etc. than control; ^b^p ≤ 0.05, etc. than Cpz, Se groups; ^c^p ≤ 0.001, etc. than Se, Cpz, Se + Cpz groups; ^d^p ≤ 0.001, etc. than OX group (n = 6 and mean ± SD). [Ca^2+^]_i_ expressed as nM. **(C)** The total amount of calcium influx was expressed as a comparative percentage between groups. **(D)** The time-dependent change in SN cells in CAP-stimulated [Ca^2+^]_i_ was expressed for all groups. **(E)** Effect of Se on TRPV1 mediated [Ca^2+^]_i_ in OX-exposed SN cells (^a^p ≤ 0.001, etc. than control group; ^b^p ≤ 0.05, etc. than Se group; ^c^p ≤ 0.001, etc. than Se, Cpz, Se + Cpz groups; ^d^p ≤ 0.05 Cpz, etc. than Se + Cpz groups and ^e^p ≤ 0.001 OX group (n = 6 and mean ± SD). **(F)** The total amount of calcium influx was expressed as a comparative percentage between groups. **(G)** The time-dependent change in MCF-7 cells in CAP-stimulated [Ca^2+^]_i_ was expressed for all groups. **(H)** Effect of Se on TRPV1 mediated [Ca^2+^]_i_ in OX-exposed MCF-7 cells (^a^p ≤ 0.001, etc. than control; ^b^p ≤ 0.001 control, etc. than Cpz and Se groups; ^c^p ≤ 0.001, etc. than Cpz, Se and Se + Cpz groups; ^d^p ≤ 0.001, etc. than OX; ^e^p ≤ 0.001, etc. than OX + Cpz and OX + Se groups (n = 6 and mean ± SD). **(I)** The total amount of calcium influx was expressed as a comparative percentage between groups.

To investigate the role of TRPV1 cation channels in OX-induced pain, after obtaining current recordings from a single cell using the patch-clamp technique, we also analysed the cell suspension (in a transparent cuvette containing at least 1 million cells) using a spectrofluorometric Fura-2 calcium signal analysis method. In this way, a comparison was made of the TRPV1 channel activation results obtained from a single cell and the cell population in the presence of OX-induced pain. The contribution of TRPV1 to the [Ca^2+^]_i_ in DRG and SN neurons, as the primary site affected by OX-induced peripheral pain, was investigated. For this purpose, specific TRPV1 channel agonists and antagonists–CAP and Cpz–were used, respectively. The curve graphs show the change in intracellular calcium concentrations due to TRPV1 activation versus time (Figures 4A, D), and the pie charts show the intergroup % [Ca^2+^]_i_ calcium ratios (Figures 4C, F). Se and Cpz decreased [Ca^2+^]_i_ more than in the control group (Figures 4B, E) (Se, Cpz and Se + Cpz decreased the intracellular calcium level significantly more than in Control group p ≤ 0.001). The peripheral sensory neurons had higher [Ca^2+^]_i_ levels than the controls in the OX groups, while Cpz (TRPV1-specific antagonist) strongly suppressed any increases in intracellular calcium concentrations (Figures 4B, E) (OX + Cpz, than in OX group p ≤ 0.001). In addition, [Ca^2+^]_i_ levels in the Se-treated group (OX + Se) were lower than in OX (p ≤ 0.001) (Figures 4B, E). The amount of [Ca^2+^]_i_ in these peripheral sensory neuron cells was lower in the OX + cpz groups than OX (p ≤ 0.001) (Figures 4B, E).

### MCF-7 cell Ca^2+^ signal analyses

Our *in vivo* experiments were validated by performing the analyses in the MCF-7 cell line (as *in vitro*), and the obtained results supported each other. A significant increase in [Ca^2+^]_i_ was observed in the OX group (p ≤ 0.001), although this situation was reversed with Se treatment (p ≤ 0.001). The Ca^2+^ influx in these cells was reduced by the TRPV1 channel inhibition of Cpz and Se to a greater degree than in the control group (p ≤ 0.001) (Figure 4H), and the co-administration of Se and Cpz nearly doubled this reduction (p ≤ 0.001). As in the other *in vivo* groups, here also the pie and curve graphs present % calcium, calcium change versus time respectively (Figures 4G, I).

### Effects of Se on cytokine levels in DRG neurons of OX-IN rats

**FIGURE 5:**
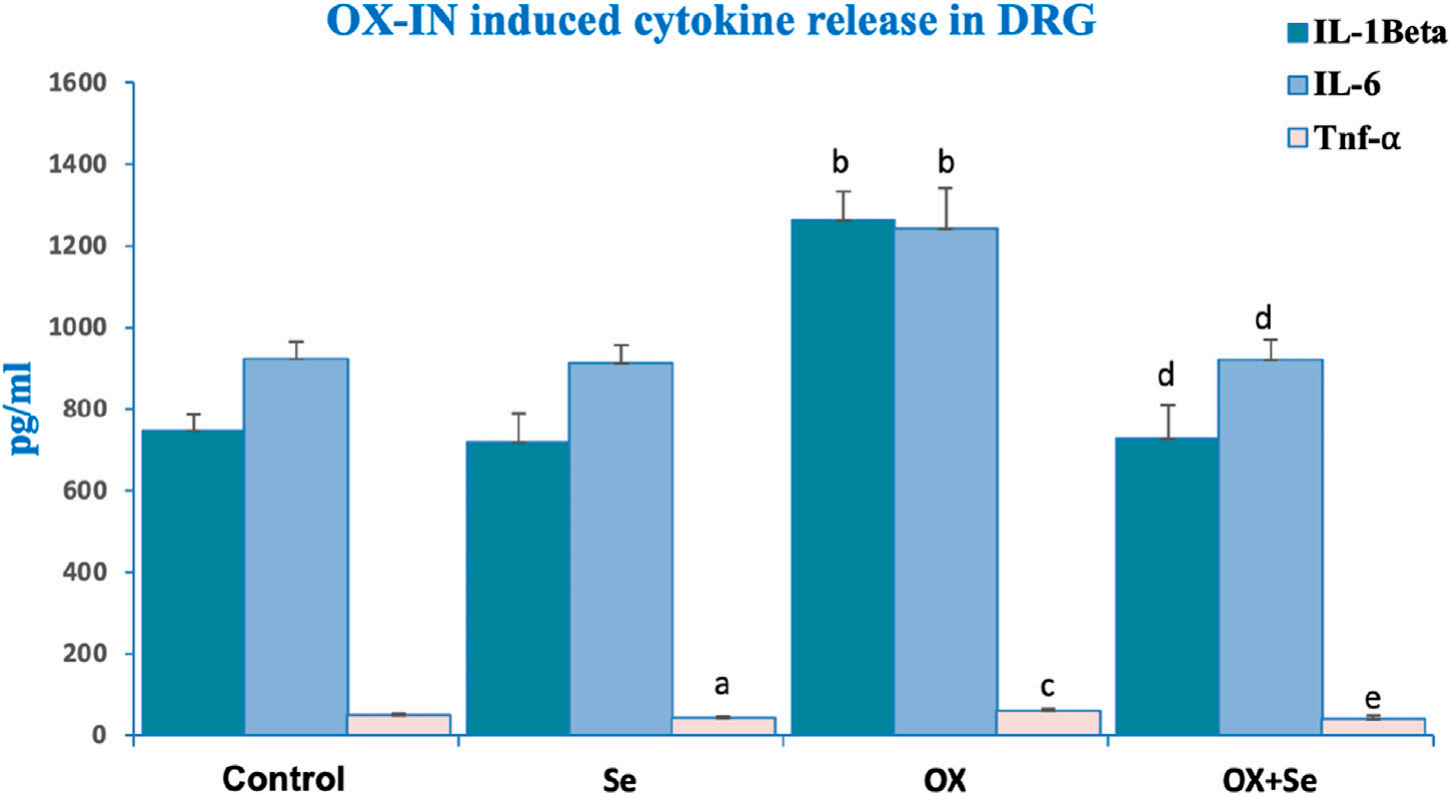
The effect of Se treatment on OX-induced cytokine release in DRG neurons. Effect of Se on IL-1 Beta secretion in OX-IN induced DRG neurons (^b^p ≤ 0.001, etc. than control and Se groups; ^d^p ≤ 0.001, etc. than OX) (n = 6 and mean ± SD). Effect of Se on IL-6 levels in OX-IN induced DRG neurons (^b^p ≤ 0.001, etc. than control and Se; ^d^p ≤ 0.001, etc. than OX) (n = 6 and mean ± SD). Effect of Se on TNF-α levels in OX-IN induced DRG neurons (^a^p ≤ 0.05, etc. than control; ^c^p ≤ 0.05, etc. than Control and Se; ^e^p ≤ 0.05, etc. than OX) (n = 6 and mean ± SD).

TNF blockers can only temporarily relieve peripheral pain through the suppression of symptoms. The cytokine levels significantly increased in the OX groups. These results were supported by simple observational pain test results. Se treatment suppressed OX-induced cytokine release. TNF-α, IL-1β and IL-6 levels were significantly increased in the OX than control and Se groups (^b^p ≤ 0.001, vs. control) and Se significantly suppressed these in OX treated groups (^d^p ≤ 0.001, ^e^p ≤ 0.05 vs. OX) (Figure 5).

### ROS, mitochondrial depolarization (JC-1) and apoptosis results in DRG neurons

**FIGURE 6:**
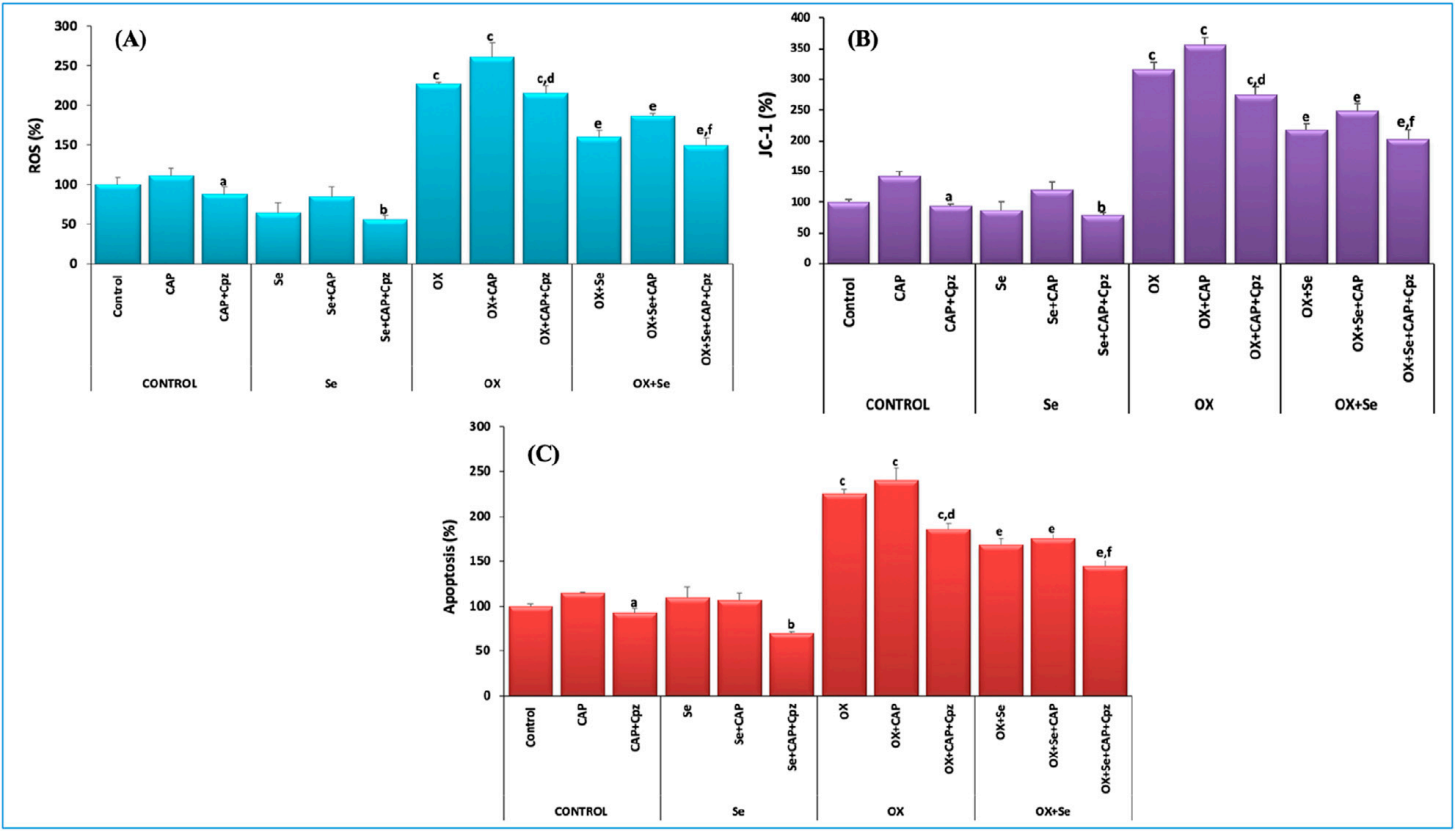
TRPV1-mediated effect of Se in OX-IN in rat DRG primary sensory neurons was evaluated for ROS, JC-1 and apoptosis analysis. **(A)** ROS results (^a^p ≤ 0.05, etc. than control-CAP group), (^b^p ≤ 0.05, etc. than Se and Se + CAP from the Se group), (^c^p ≤ 0.001, etc. than control and Se groups; OX ^d^p ≤ 0.05, etc. than CAP (^e^p ≤ 0.05, etc. than OX group; ^f^p ≤ 0.05, etc. than OX + Se and OX + Se + CAP in OX + Se group) (n = 6 and mean ± SD). **(B)** JC-1 results (^a^p ≤ 0.05, etc. than CAP from the control group), (^b^p ≤ 0.05, etc. than Se group), (^c^p ≤ 0.001, etc. than control and Se groups; ^d^p ≤ 0.05, etc. than CAP (^e^p ≤ 0.05, etc. than OX; ^f^p ≤ 0.05, etc. than OX + Se and OX + Se + CAP in OX + Se group) (n = 6 and mean ± SD). **(C)** Apoptosis results (^a^p ≤ 0.05, etc. than control-CAP), (Se from Se group and Se + CAP, ^b^p ≤ 0.05), (^c^p ≤ 0.001, etc. than control and Se; ^d^p ≤ 0.05, etc. than OX + CAP (^e^p ≤ 0.05, etc. than OX; ^f^p ≤ 0.05, etc. than OX + Se and OX + Se + CAP in OX + Se group) (n = 6 and mean ± SD).

Excessive and persistent increases in intracellular calcium concentrations–an important secondary messenger in all physiological events–is an important cause of mitochondrial oxidative stress that leads to cell death. Excess intracellular calcium depolarizes the double-membrane mitochondrial membrane and causes cytochrome c release into the cytosol. This creates caspase 9 and caspase 3 by triggering caspase cascade reactions, leading to apoptosis of the cell. In this study, we investigated OX-induced oxidative stress and the protective effect of Se (from mitochondrial depolarization to apoptosis), and the results were quite impressive. OX-induced ROS, mitochondrial depolarization (JC-1) and apoptosis levels increased more than in the controls (p ≤ 0.001), while these values were more significantly suppressed in the Se + OX and Se + Cpz groups than in the OX group in the DRG neurons (p ≤ 0.05) (Figure 6).

### ROS, mitochondrial depolarization (JC-1) and apoptosis in MCF-7 cell line

**FIGURE 7:**
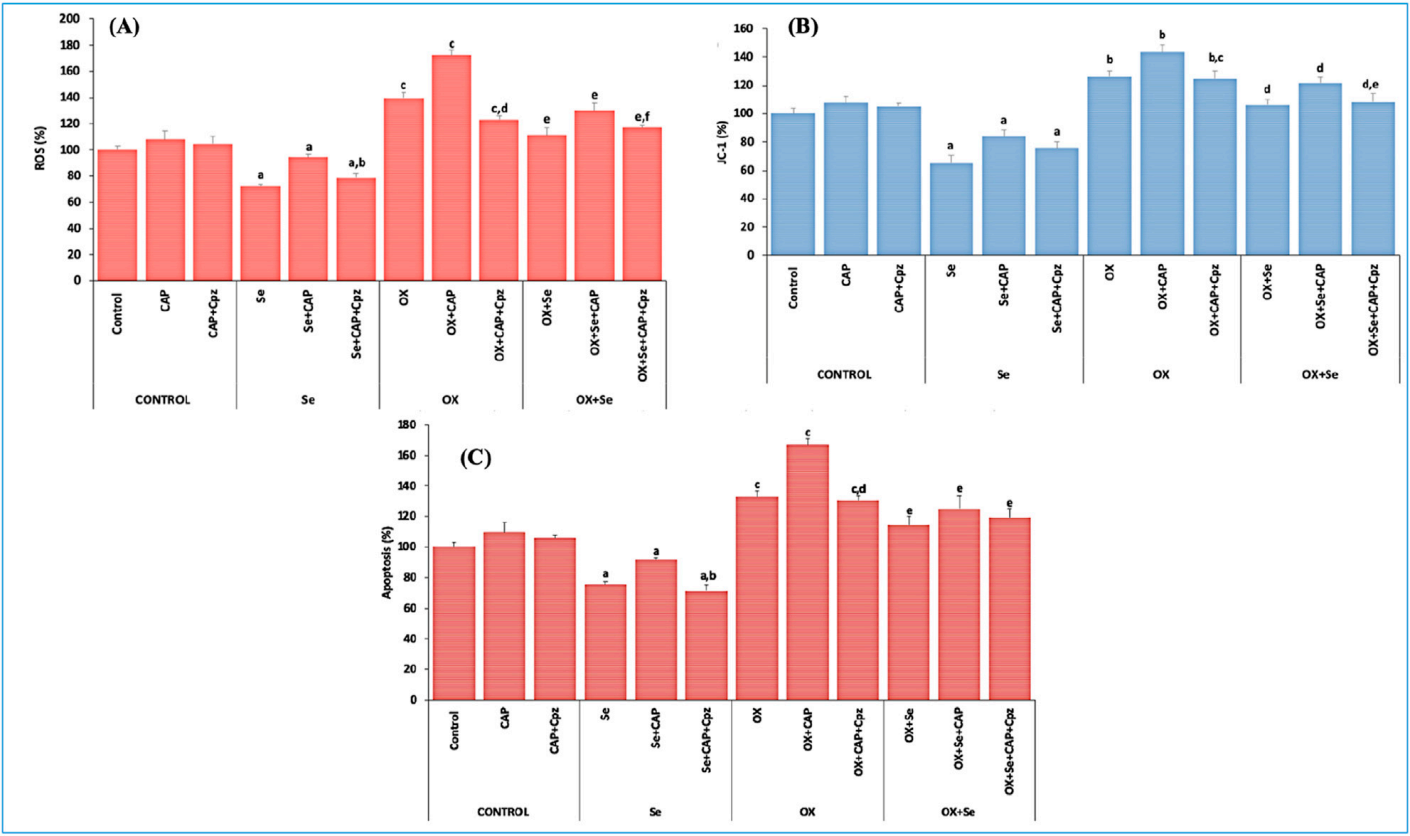
The effect of TRPV1-mediated Se in MCF-7 breast cancer cells exposed to OX was evaluated for ROS, JC-1 and apoptosis analysis. **(A)** ROS results (^a^p ≤ 0.05, etc. than control group; ^b^p ≤ 0.05, etc. than Se + CAP) (^c^p ≤ 0.01, etc. than control and Se groups; ^d^p ≤ 0.01, etc. than OX + CAP), (^e^p ≤ 0.05, etc. than OX group; ^f^p ≤ 0.05, etc. than OX + Se + CAP group), (n = 6 and mean ± SD). **(B)** JC-1 results (^a^p ≤ 0.05, etc. than control group), (^b^p ≤ 0.05, etc. than control and Se groups; ^c^p ≤ 0.05, etc. than OX + CAP), (^d^p ≤ 0.05, etc. than OX group; ^e^p ≤ 0.05, etc. than OX + Se + CAP (n = 6 and mean ± SD). **(C)** Apoptosis results (^a^p ≤ 0.05, etc. than control; ^b^p ≤ 0.05, etc. than Se + CAP) (^c^p ≤ 0.01, etc. than control and Se groups; ^d^p ≤ 0.01, etc. than OX + CAP), (^e^p ≤ 0.05 compared to OX group) (n = 6 and mean ± SD).

We repeated the *in vitro* apoptosis experiments using the MCF-7 cell line, and the obtained results concurred with the *in vivo* values. ROS, mitochondrial membrane depolarization (JC-1) and apoptosis levels were higher in the OX groups compared to the control (p ≤ 0.001). These values were significantly suppressed in the Se + OX and Se + Cpz groups (p ≤ 0.05) (Figure 7).

### Effect of Se on lipid peroxidation and glutathione peroxidation values in OX-IN

The effects of Se and OX on lipid peroxidation glutathione and glutathione peroxidase values in DRG neurons are shown in Table 1. Se, an important trace element, is thought to have a protective effect against ROS as a powerful antioxidant (Kahya et al., 2017), although this role of Se in OX-induced peripheral pain has yet to be studied. The results obtained in the present study were impressive. The lipid peroxidation (LP) levels in OX-treated rat DRG neurons were observed to be higher than in the control group (p ≤ 0.01), and a reduction was observed in GSH-Px values (p ≤ 0.05). GSH levels (p ≤ 0.01) and GSH-px levels (p ≤ 0.05) were higher in DRG of the Se-treated rat than in the controls, while LP levels were decreased (Table 1) (p ≤ 0.05).

**TABLE 1:** Lipid Peroxidation (LP) levels, reduced glutathione (GSH) and glutathione peroxidase (GSH-Px) activities in DRG neurons (Control, Se, OX and OX + Se groups) (^a^p ≤ 0.05 and ^b^p ≤ 0.01, etc. than control, ^c^p ≤ 0.05 and ^d^p ≤ 0.01, etc. than OX).

Parameters	Control	Se	OX	OX + Se
LP (μmol/g protein)	19.83 ± 1.72	16.05 ± 1.94^a^	26.78 ± 2.08^b^	22.67 ± 1.36^c^
GSH (μmol/g protein)	14.92 ± 1.20	21.39 ± 1.20^b^	14.50 ± 0.67	16.51 ± 1.10^c^
GSH-Px (IU/g protein)	12.71 ± 6.66	16.76 ± 6.31^a^	9.36 ± 6.09^a^	15.12 ± 6.82^d^

## Discussion

The results of the present study demonstrated that Se treatment could reduce OX-IN-induced pain intensity, [Ca^2+^]i accumulation, mitochondrial ROS, and apoptosis levels in both DRG and SN through the inhibition of TRPV1 channels. This study provides the first evidence to investigate OX-IN pathophysiology focusing on the roles of TRPV1 channels *in vivo* (in central and peripheral pain in DRG and SN) and *in vitro* (MCF-7 cells). OX is a highly successful chemotherapeutic agent in cancer treatment, although the neurotoxicity that develops in peripheral neurons following treatment is a side effect that limits its therapeutic potential. The main mechanisms of this neurotoxicity are still controversial. The present study investigated the roles of TRPV1 cation channels, which are highly expressed in DRG neurons (Caterina et al., 1997; Akpinar et al., 2016; Kahya et al., 2017) in OX-IN peripheral neuropathy, and focused on the basic mechanisms of this neurotoxicity. DRGs are essential for understanding the molecular basis of neuropathic pain (Yüksel et al., 2017; Park and Kiernan, 2018). In the present study, OX caused peripheral neuropathic pain, and Se significantly prevented this OX-induced pain. Thermal and mechanical peripheral pain during chemotherapy is a distinctive feature of Chemotherapeutic pain (Ertilav et al., 2021). Numerous pain test methods are used to evaluate chemotherapy induced pain. Among them, von Frey and hot plate test models are two important and common models to evaluate chemotherapy-induced mechanical and thermal hyperalgesia (Yüksel et al., 2017). According to the results of observational pain testing, a significant decrease in pain threshold was detected following 1 week of OX administration, and this decrease was reversed by Se. Moreover, the decrease in these values continued gradually in the following weeks. Although thousands of studies have been conducted on OX-induced pain (Liu et al., 2018), the TRPV1-mediated neuroprotective effects of Se have not yet been examined in OX-IN.

Impaired calcium homeostasis has been reported in OX-IN mediated neurotoxicity (Anand et al., 2010). One of the calcium-permeable cationic channels is the TRP superfamily discovered in the photoreceptors of *Drosophila* vinegar flies (Montell and Rubin, 1989). TRPV1, belonging to the vanilloid subfamily of this group, is highly expressed in the nervous system and its roles in the pathophysiology of OX-IN remains unclear (Xu et al., 2024; Nazıroglu and Braidy, 2017). The Se-mediated contribution of TRPV1 to impaired calcium homeostasis in OX-induced neurotoxicity has not yet been elucidated. TRPV1 channels, attracted our attention due especially to their production of free oxygen radicals and their contribution to cell death due to inflammation (Xiang et al., 2017). TRPV1 channels are known to be permeable to calcium ion and sensitive to oxidative damage and have undeniably contributed to many pain studies (Ogawa et al., 2016; Hu et al., 2019). The current study demonstrates the Se-mediated role of TRPV1in the pathogenesis of OX-IN by patch-clamp recordings. The significant increase in current density observed in the OX group was severely inhibited in the OX + Se groups, while Cpz significantly reduced current densities in both the control and OX groups. The current recordings obtained from DRG neurons in a whole cell configuration and the calcium signal analysis results (in DRG, SN and MCF-7 cells) showed that Se effectively suppressed TRPV1 overactivity in *in vivo* and *in vitro* OX-IN models. The antagonistic effects of Se (like Cpz) on TRPV1 channels support our findings (Balaban et al., 2017; Kahya et al., 2017). These results clearly demonstrate the role of Se via TRPV1 channels in OX-IN peripheral pain.

Impaired Ca^2+^ influx through TRPV1 and voltage-gated Ca^2+^ channel activation triggers mitochondrial depolarization in sensory neurons (Pabbidi et al., 2008). This excessive mitochondrial depolarization leads to increased ROS production and disrupts Ca^2+^ homeostasis, particularly affecting voltage-gated Ca^2+^ channels (Naziroglu et al., 2012a). In this study, the effect of Se on oxidative stress, from mitochondrial membrane depolarization to apoptosis, in relation to TRPV1, was investigated using molecular techniques. Apoptosis levels, ROS and mitochondrial membrane depolarization levels in DRG neurons were significantly increased in the OX group. Se suppressed the abnormal increases caused by OX and positively affected cell viability. The Cpz treatment of these neurons significantly reduced the oxidative cytotoxicity induced by OX. We also evaluated the effect of Se on apoptosis, ROS and JC-1 assays in MCF-7 cells exposed to OX *in vitro*, and our results confirmed our *in vivo* results. It appears that Se treatment reduces OX-IN-induced mitochondrial ROS in DRG and SN, and that OX-IN-induced apoptosis is reversed by Se treatment. Therefore, the present results suggest that Se may modulate OX-IN-induced cellular oxidative stress in neurons. In addition, glutathione peroxidase levels were found to decrease and malondialdehyde level (LP) to increase significantly in DRG neurons in the OX group. The glutathione and glutathione peroxidase values increased in the groups in which Se and OX were applied together, while the lipid peroxidation level was severely suppressed. This neuroprotective effect of Se–a trace element–may be attributed to its antioxidant properties (Rayman, 2000; Kahya et al., 2017). Our results are consistent with those of other studies on peripheral oxidative stress tests on OX-IN in literature (Joseph et al., 2008). Antioxidants such as GSH and GSH-Px play a critical role in suppressing ROS production in mitochondria (Flatters, 2015; Lawrence and Burk, 1976). Therefore, GSH, GSH-Px, and related enzymes are believed to be essential in protecting cells from ROS damage. Se acts as a co-factor for the GSH-Px enzyme, and selenium modulates mitochondrial respiratory chain reactions (Schweizer et al., 2004). In many experimental models, Se treatment has been shown to increase GSH and GSH-Px levels (Uğuz and Nazıroğlu, 2012). Additionally, the significantly increased TNF-α, IL-1β, and IL-6 levels in the DRGs of OX-treated animals were ameliorated by Se treatment. Selenium, in the form of selenocysteine in humans, functions as a redox center that reduces the spread of oxidative damage to lipids, lipoproteins, and DNA (Naziroglu et al., 2012b). Adequate selenium intake is important for protecting against cellular inflammation (Brozmanová et al., 2010). Selenium salts have been reported to inhibit the production of inflammatory cytokines, including IL-1α, interleukin-6, and TNF-α (Celerier et al., 1995). This clarifies the role of selenium in reducing oxaliplatin-induced oxidative stress (Balaban et al., 2017).

## Conclusions

In conclusion, promising results have been obtained in preventing OX-IN, but clinical evidence supporting its efficacy is limited. Current treatments for OX-IN primarily address symptoms, and therefore, molecular-based approaches are needed. There are currently several therapeutic drugs available for the treatment of OX-induced pain that can temporarily reduce neuronal hyperexcitability by preventing pain conduction. The success of duloxetine–as one such drug–allows patients to be classified according to the chemotherapeutic agent that triggers neuropathy (Smith et al., 2013; Ertilav et al., 2020). However, such drugs do not inhibit microgliosis or inflammatory responses and thus cannot be used for the treatment of oxaliplatin-induced pain (Smith et al., 2013). In the future, the development of double-acting drugs that act via the TRP channels and reduce cytokine release will be necessary for the treatment of OX-IN. Based on the results of this study, selenium shows promise in its protective effect against OX-IN pain mediated by TRPV1 channels.

## Data Availability

The raw data supporting the conclusions of this article will be made available by the authors, without undue reservation.
